# External and internal triggers of cell death in yeast

**DOI:** 10.1007/s00018-016-2197-y

**Published:** 2016-04-05

**Authors:** Claudio Falcone, Cristina Mazzoni

**Affiliations:** Pasteur Institute-Cenci Bolognetti Foundation; Department of Biology and Biotechnology “Charles Darwin”, Sapienza University of Rome, Piazzale Aldo Moro 5, 00185 Rome, Italy

**Keywords:** Yeast, Apoptosis, Caspase, Autophagy, Necrosis

## Abstract

In recent years, yeast was confirmed as a useful eukaryotic model system to decipher the complex mechanisms and networks occurring in higher eukaryotes, particularly in mammalian cells, in physiological as well in pathological conditions. This article focuses attention on the contribution of yeast in the study of a very complex scenario, because of the number and interconnection of pathways, represented by cell death. Yeast, although it is a unicellular organism, possesses the basal machinery of different kinds of cell death occurring in higher eukaryotes, i.e., apoptosis, regulated necrosis and autophagy. Here we report the current knowledge concerning the yeast orthologs of main mammalian cell death regulators and executors, the role of organelles and compartments, and the cellular phenotypes observed in the different forms of cell death in response to external and internal triggers. Thanks to the ease of genetic manipulation of this microorganism, yeast strains expressing human genes that promote or counteract cell death, onset of tumors and neurodegenerative diseases have been constructed. The effects on yeast cells of some of these genes are also presented.

## Introduction

Regulated cell death (RCD) can be executed through distinct subroutines, sometime partially overlapping, leading to apoptosis, autophagy and programmed necrosis [1], and such scenarios have been described also in yeast cells under physiological or induced stress conditions.

In mammals, RCD can be observed during aging or in consequence of pathologies including neurodegenerative disorders, hypoxia and heart strokes. In the opposite direction, loss of RCD can result in the onset of cancer.

Due to its simple handling, yeast represents a very useful eukaryotic model system for deciphering the complex network of the different and often interwoven RCD pathways occurring in mammals, aiming, in particular, to the identification of executors, to the role of cellular organelles and compartments, and to the detection of new cell phenotypes in response to external and internal triggers.

## Apoptotic cell death

Yeast apoptosis was first described in cells carrying a mutated *CDC48* gene (cdc48^S565G^), which codes for the AAA-ATPase and has roles in cell division, ubiquitin-dependent ER-associated protein degradation (ERAD) and vesicle trafficking [2]. Later on, it was found that mutations in the VCP gene, the metazoan homolog of the yeast *CDC48*, gave rise to apoptotic phenotypes in mammalian cell cultures [3, 4], in *trypanosomes* [5], in *Drosophila* [6] and in zebrafish [7]. Like mammalian cells, yeast cells undergoing apoptosis display characteristic markers such as DNA breakage, chromatin condensation, phosphatidylserine externalization, reactive oxygen species (ROS) accumulation and cytochrome *c* release from mitochondria. In nature, this process might favor the elimination from the yeast population of old and/or unhealthy cells, increasing the availability of nutrients for young and healthy cells [8].

In this organism, apoptosis is induced by internal and external triggers including cellular dysfunctions, H_2_O_2_, acetic acid and many others [9, 10]. Although lacking Bax and Bcl genes, several yeast orthologs of mammals’ main apoptotic regulators, such as *AIF1* (AIF), *NUC1* (EndgoG), *MCA1/YCA1* (metacaspase), *NDI1* (AMID), *NMA111* (HtrA2/Omi) and others, have been identified, demonstrating that the basal apoptotic machinery is present in this unicellular organism [11].

## Necrotic cell death

Necrosis in mammals is a physiological cellular process that becomes more evident in some disorders and after virus and bacterial infection. In contrast to apoptosis, necrotic cells release intracellular contents following the plasma membrane rupture. In yeast cells, H_2_O_2_, acetic acid and heavy metals, well-known triggers of apoptosis at low doses, can also induce accidental necrosis at higher concentration because of the excessive damage to cellular components [9, 10, 12].

Yeast cells also posses a programmed necrotic pathway under conditions similar to those regulating programmed necrosis in mammals [1]. Necrosis in yeast is positively regulated by aging, low pH and mitochondria while inhibited by spermidine, EndoG, vacuolar and peroxisomal functions [13]. Homologs of known mammalian mediators of necrosis have been found in the *Saccharomyces cerevisiae* genome but additional studies are still needed to identify the executors and clarify a putative altruistic meaning of necrotic cell death in unicellular yeasts. Liponecrosis has been recently reported as an additional cell death module of RCD in yeast cells exposed to exogenous palmitoleic acid (POA) [14]. Cells undergoing liponecrosis do not show hallmarks of apoptosis nor plasma membrane rupture observed in necrosis and exhibit, as in autophagic cell death, a non-selective degradation of cellular organelles but not increased cytoplasmic vacuolization. Peroxisomal fatty acid oxidation acts as a pro-survival process in that protects yeast cells from liponecrotic death by reducing the cellular level of POA [14]. Deletion of *ATG32*, the gene encoding a mitochondrial outer membrane protein required to initiate mitophagy, results in increased sensitivity to death triggered by POA, pointing to macromitophagy as an opponent of POA-induced cell death. Interestingly, the absence of the serine/threonine kinase Atg1p, which is required for the execution of macroautophagy, while lowering clonogenic survival of yeast cells submitted to apoptosis by H_2_O_2_, reduces the lethality induced by POA [14]. Moreover, while Nuc1p, Aif1p and Nma111p are not involved in POA response, the absence of the metacaspase Mca1p enhances POA-induced cell death, suggesting a pro-survival role of this apoptotic regulator [15].

These results altogether reinforce the idea that POA-triggered death is in fact an additional new form of RCD.

## Autophagy and cell death

Macroautophagy is a conserved process required for the degradation and recycling of cytoplasmic components by delivering molecules and entire organelles to the vacuole or to lysosomes, depending on the eukaryotic cell type. The morphological hallmark of autophagy is the formation of the autophagosome, a vesicle bounded by a double membrane, within which cytoplasmic components remain randomly (non-selective autophagy) or selectively (selective autophagy) sequestered. Selective autophagy ensures the health of cells by removing protein aggregates and carrying the turn-over of mitochondria (mitophagy), peroxisomes (pexophagy), lipids (lipophagy), and ER (ER-phagy), which might represent potential triggers of apoptosis. After fusion with the vacuole, the material is degraded to simpler molecules, which are then released again into the cytoplasm for reuse. Autophagy, as well selective autophagy [16], occurs in yeast cells at basal level and is up-regulated by nutritional changes and starvation. The regulation of the process determines the size and number of autophagosomes under normal and inducing conditions. Studies with yeast allowed the identification of more than 30 autophagy-related (Atg) proteins, most of which are conserved in higher eukaryotes. In the presence of nutrients, most of *ATG* genes are repressed at transcriptional level in consequence of the inhibition of activators and/or activation of repressors of autophagy [17]. In several organisms, under specific conditions, autophagy mediates a particular type of RCD, defined as autophagic cell death [18]. In yeast, relationships between autophagy and cell death are still to be explored, and some evidences suggest that autophagy may accelerate cell death in *S. cerevisiae* following the expression of human p53, BAX and under starvation conditions [19–21].

## Yeast cell death regulators

One of the first genes involved in yeast RCD was *MCA1/YCA1*, which codes for a protein showing a caspase activity and plays an important role in regulating apoptosis in yeast [22]. Mca1p has been classified as a type I metacaspase, found also in many other organisms, with a characteristic N-terminal pro-domain that is thought to be cleaved off upon activation [23]. The autocatalytic processing of Mca1p requires Ca^2+^ ions and the crystal structure of the protein revealed a canonical caspase-like fold and it can exist as a monomer in both solution and crystals [24]. While caspases cleave substrates after an aspartate residue, metacaspases cleave after arginine or lysine and the identification of their substrates is poorly known [25]. Concerning the Mca1p substrates, so far only the glycolytic enzyme GAPDH (glyceraldehyde 3-phosphate dehydrogenase) has been identified as a possible target, although the physiological relevance and effects of this cleaved protein remain to be elucidated [26]. Mca1p has also been shown to be involved in protein quality control (PQC) displaying some pro-survival functions, which suggest a dual role of this protein acting in both pro-death and pro-survival pathways [27–29]. As an example, although an increased caspase activity was detected in the *cdc48*^*S565G*^ mutant [30], from a synthetic genetic array (SGA) analysis it was found that a *cdc48* conditional mutant negatively interacted with the *mca1* null mutant, suggesting that Mca1p can buffer the absence of Cdc48p [27]. It has been estimated that about 40 % of cell death in yeast is Mca1p dependent, suggesting the presence of many alternative cell death pathways. Beside Mca1p, there are other proteases involved in yeast PCD. The caspase-like protease Esp1p, upon H_2_O_2_ cell exposure, cleaves cohesin Mcd1/Rad21. The truncated C-terminal fragment of Mcd1p translocates from the nucleus to mitochondria, causing the decrease of mitochondrial membrane potential and the release of cytochrome *c* [31]. Moreover, the protease activity Kex1p plays a role in promoting yeast PCD in *wbp1*-*1* N-glycosylation mutants, during chronological aging, following tunicamycin and acetic acid treatments [32].

Apoptosis-inducing factor (AIF1), homologue to mammalian AIF, is a mitochondrially localized protein that, upon apoptotic insults, translocates to the nucleus where it mediates chromatin condensation and DNA degradation [33]. As its mammalian counterpart, AIF1 DNA degradation activity requires cyclophilin A and plays a vital role in maintaining mitochondrial activity [34, 35]. *NDI1*, which codes for the inner mitochondrial membrane NADH dehydrogenase and catalyzes the oxidation of intramitochondrial NADH, is the human homologue of the AIF-homologous mitochondrion-associated inducer of cell death (AMID). In facts, *NDI1* overexpression causes cell death while its deletion lowers ROS production and extends CLS [36]. Similar effects, although to a lower extent, were observed for Nde1p, the protein localized on the outer mitochondrial membrane and responsible for oxidation of cytosolic NADH [36].

*NUC1*, the yeast ortholog of mammalian endonuclease G (EndoG), is another cell death effector that translocates from mitochondria to nucleus upon apoptosis induction [37]. Nuc1p pro-apoptotic activity requires karyopherin Kap123p, homologue to the mammalian mitochondrial permeability transition pore (MPTP), and H2B phosphorylation.

The absence of *NUC1* protects yeast from cell death only in respiratory conditions while, in fermentative conditions, it enhances necrotic cell death [37]. EndoG has a vital role in both yeast and mammalian cells in maintaining polyploidy cells [38]. Nma111p (nuclear mediator of apoptosis) is a yeast serine protease, homologous to the mammalian pro-apoptotic protein mitochondrial-located HtrA2/Omi that, differently from the latter, it resides in the nucleus. Deletion or overexpression of *NMA111* causes protection or induction of cell death, respectively [39].

Bir1p, the target of the Nma111p activity, acts as an inhibitor of apoptosis (IAP) in yeast [40].

Pep4p, a pepsin-like aspartic protease ortholog of human Cathepsin (CatD), translocates from the vacuole to the cytosol and is involved in the degradation of nucleoporins following H_2_O_2_-induced apoptosis [41]. Yeast *pep4* mutant strains undergo both apoptosis and necrosis during chronological aging.

The proteolytic activity of Pep4p is required to contrast apoptotic cell death while the non-proteolytic part of this protein is involved in its anti-necrotic function [13]. In addition Pep4p, as well as CatD, shows a protective role in acetate-induced apoptosis in both yeast and colorectal cancer (CRC) cells [42, 43] depending on its proteolytic capacity. In mammalian cells, BH3-only proteins upon induction of apoptosis are targeted to mitochondria where they induce mitochondrial outer membrane permeabilization (MOMP), thereby initiating the regulated disintegration of the cell [44, 45]. The presence of a BH3-only protein encoded by the *YNL305c* gene has been reported in yeast, later renamed *YBH3*. Overexpression of Ybh3p sensitizes yeast to apoptotic stimuli, while its absence protects cells against H_2_O_2_, acetic acid, murine BAX expression and extends both replicative and chronological lifespan. Ybh3p-mediated cell death is independent of Mca1p, Aif1p, Nma111p and Nuc1p, suggesting that Ybh3p triggers its own mitochondrial cell death pathway [46]. The same gene, named *BXI1* (for Bax inhibitor) in this occasion, has been reported to be involved in unfolded protein response (UPR) and to play a protective role in heat shock response, ethanol and glucose induced cell death [47]. The opposite pro- and anti-apoptotic role of this protein might depend on the apoptosis-inducing conditions applied.

## Internal triggers

Alterations of fundamental cellular pathways, such as DNA replication, actin cytoskeleton, mRNA stability and protein modifications and degradation, produce triggers that induce various forms of yeast cell death [48–53].

Some forms of cell death require the activity of the metacaspase encoded by the *MCA1* gene [22]. Differently, other forms of PCD are independent of Mca1p and follow alternative pathways. Yeast cell death can also be induced by expressing heterologous genes involved in apoptosis, tumor suppression and neurodegenerative diseases.

### Reactive oxygen species (ROS)

Yeast cells accumulating ROS show morphological markers peculiar for apoptosis in mammals. The phenomenon is caspase dependent in that, in the absence of the metacaspase Mca1p, apoptosis is prevented [22].

ROS in yeast are mainly produced within mitochondria [54] during aerobic respiration as a result of the leakage of electrons from the respiratory chain, which react with oxygen. When ROS concentration exceeds normal antioxidant defenses, yeast cells respond with the activation of transcription factors, namely Yap1p, Skn7p, Msn2p, and Msn4p that, in turn, regulate transcription of genes involved in antioxidant defenses [55, 56]. ROS accumulation is the cause of oxidative damaging of nucleic acids, proteins and lipids and, depending on concentration, can induce apoptosis as well necrosis in different cells. ROS-induced cell death has been put in relation with aging, neurodegenerative diseases and cancer and many articles have focused on oxidative stress also in yeast [55, 57].

### Aging

Replicative life span (RLS) and chronological life span (CLS) are two models of aging in yeast cells. RLS refers to the number of divisions of an individual cell before budding arrest while CLS represents the time a culture maintains viability during stationary phase. In both cases, cell death eliminates old and damaged cells and is accompanied by typical markers of apoptosis [58–60]. Nevertheless, the mechanisms underlying the two forms of cell death are only partially overlapping. As an example, the deletion of the metacaspase gene *MCA1* prevents apoptotic cell death during CLS while, in contrast, reduces the numbers of cell divisions in RLS [27, 59]. During CLS, in addition to apoptosis, yeast cells die also through programmed necrosis [11].

### DNA replication and RNA stability

Initiation of DNA replication is a conserved process that requires the origin recognition complex (*ORC*), constituted by a six-subunit complex of proteins [61]. *ORC2* codes for a subunit of the *ORC* complex and *orc2*-*1 ts* mutant cells, at non-permissive temperature, show defects in initiation of DNA replication, activate DNA damage responses, premature aging and undergo apoptosis following the Mca1p-dependent pathway [53, 62].

An important component of the pre-replicative complex required for the initiation of eukaryotic DNA replication is Cdc6p, which is rapidly degraded in a proteasome-dependent way in cells undergoing apoptosis induced by the DNA-damaging drug adozelesin [63].

The steady-state level of mRNAs is another fundamental cellular process, which depends on the equilibrium between mRNA synthesis and decay [64]. Mutants in decapping, a crucial event of mRNA degradation, such as *dcp1, dcp2, dhh1, lsm1*–*7*, show apoptotic phenotypes and accelerated chronological aging [51, 65]. At least in one of these mutants (*Kllsm4Δ1*), apoptotic death was demonstrated to be Mca1p dependent [66], and most of the apoptotic phenotypes were suppressed by the overexpression of *HIR1*, *PGK1* and *NEM1*, the genes encoding a subunit of the HIR complex involved in histone gene transcription, the phosphoglycerate kinase and the catalytic subunit of Nem1p-Spo7p phosphatase holoenzyme that regulates Pah1p (lipin) activity, respectively, [67–69]. This suggests a link between cell death induced by defects in RNA degradation and histone expression, ATP production and phospholipid homeostasis.

Metacaspase-dependent apoptotic cell death has been recently reported in strains defective in cytoplasmic exosome function (*ski2∆* mutants) or deadenylation (*ccr4∆pan2∆* mutants) pointing to mRNA degradation as a relevant pathway involved in yeast cell death [70].

In mammalian cells undergoing classical apoptosis, 3′ truncated mRNA decay intermediates with uridylate-rich tails are generated, which have been suggested to represent an early apoptotic phenotype [71, 72].

Although uridylyl transferase has not been described in budding yeast [73], the accumulation of capped mRNAs in decapping mutants could represent for yeast cells an internal apoptotic signal whose molecular mechanisms still need a deeper investigation.

Apoptotic stimuli in yeast also induce endonucleolytic degradation of rRNA, more evident in fermenting cells, which is dependent on histone H2B modifications and independent of Mca1p and Aif1p [74]. Upon oxidative stress, also cytoplasmic tRNAs are degraded by the release from the vacuole of the Rny1p RNase; although little is know about the cell death-induced pathway, overexpression of *RNY1* exacerbates the *bir1* and *yap1* growth defect [75]. Altogether, these results clearly point to crosstalk between RNA metabolism and RCD.

### Epigenetic modifications

Histone modifications, besides their regulative role in a variety of cellular processes such as gene transcription, DNA repair, mitosis, meiosis, and development, have been also associated to the establishment of cell death in both mammalian and yeast cells [76].

In yeast, H2B-K11 deacetylation, operated by the histone deacetylase (HDAC) Hos3p, is necessary to allow the phosphorylation of the apoptotic mark H2B-S10 (H2B-S16 in human) by Ste20p kinase, the yeast homolog of mammalian Mst1 kinase [77, 78]. Since H2B-S10 phosphorylation is also present during meiosis, it has been proposed that this modification is associated to large-scale changes in chromatin structure. [79].

Histone H2B ubiquitination is required for nucleosome stability and its loss, in the E3 ubiquitin-ligase *bre1* and ubiquitin protease *ubp10* mutants, sensitizes metacaspase-dependent cell death [80, 81].

H2B ubiquitination is a prerequisite for histone H3K4 and H3K79 methylation and it was recently reported that loss of H3K4 methylation, due to the inactivation of the methyltransferase Set1p, triggers apoptotic cell death partially dependent on Mca1p during chronological aging and on EndoG/Nuc1p after hydrogen peroxide treatment [82].

### Death in aging colonies

In liquid media, yeast population develops in the form of single cells that replicate several times and finally die through the apoptotic process. During growth on solid media, both in nature and in laboratories, cells remain in contact giving rise to colonies that enlarge over time. In this case, apoptotic death occurs in oldest cells, located in the middle of the colony, through a Mca1p or Aif1p-independent pathway in response to an ammonia signal emitted by aged surrounding colonies. Nevertheless, D2R staining of cells from colonies of the *mca1* mutant, indicated the presence of Asp-ase or another caspase-like activity [83].

## Heterologous expression of cell death genes

Expression in yeast of the human key apoptotic inducer Bax leads to apoptotic cell death accompanied by cytochrome *c* release. These events can be reverted by the contemporary heterologous expression of Bcl-2 or Bcl-xL, as well of 14-3-3β/α and human LDH [84–86].

Yeast models expressing neurotoxic proteins have been recently reviewed [87].

Expression in yeast of α-synuclein, an intracellular trigger of Parkinson’s disease, results in apoptosis as well as necrosis, which are modulated by the activities of the ubiquitin–proteasome system (UPS), autophagy, and ubiquitin-dependent vesicular trafficking [87–89]. Moreover, alpha-synuclein toxicity depends on chronological aging, functional mitochondria and it requires the presence of Nuc1p [90].

Expanded poly-glutamine (poly-Q) domains cause protein aggregation and neurodegeneration [91, 92]. Although the yeast genome does not contain an ortholog of the human gene htt, responsible for Huntington disease (HD), expression of Htt103Q in yeast leads to widespread cellular dysfunction resulting in death with apoptotic markers [92]. Poly-Q aggregates induce ER stress, mitochondrial dysfunction and impair vesicle-based protein degradation, including autophagy [87].

The spinocerebellar ataxia type-3 (SCA3) is another disease caused by the expansion of poly-Q in the gene atxn3, which encodes a protein known as ataxin-3 (AT3) [93]. The expression of AT3 in yeast is toxic and leads to accumulation of ROS, imbalance of the antioxidant defense system, loss in cell membrane integrity and necrotic cell death, without the induction of apoptosis [94].

Expression in yeast of a mutated form of DFNA5, a gene responsible for autosomal dominant hearing loss (HL), induces ROS accumulation leading to a caspase-independent cell death that relies on mitochondrial functions such as the mitochondrial fission protein Fis1p, the voltage-dependent anion channel Por1p, and the mitochondrial adenine nucleotide translocators Aac1p and Aac3p [95]. More recently, it has been reported that ER and protein folding are also involved in RCD caused by DFNA5 both in yeast and in HEK293T cells [96]. It has also been reported that the heterologous expression of human immunodeficiency virus (HIV-1) protease [97] and the proteinaceous elicitor harpin (Pss) from *Pseudomonas syringae* [98] led to regulated necrosis in budding yeast.

Yeast has also been used as a model for the study of tumors, also because of similarities in carbon metabolism with cancer cells. The p53 tumor suppressor protein is a nuclear phosphoprotein that plays a key role in safeguarding genome integrity [99]. Mutations in the p53 gene are found in 45 % of human cancers cells that, in consequence, undergo uncontrolled cell proliferation and often acquire resistance to chemotherapy [99]. *S. cerevisiae* does not contain p53 homologues and, in this respect, it can be considered a powerful ‘clean room’ model to study the different molecular pathways associated with the presence of this protein. Mammalian p53 can function as a transcription factor in yeast [100] and several groups used yeast for studying the transcriptional activity of human wild-type and tumor-derived p53 mutated proteins [101]. More recent studies have shown that the expression of p53 induces apoptosis-like cell death making p53, after Bax, the second heterologous pro-apoptotic gene, and strengthening the idea that the apoptotic processes are conserved throughout the evolution [102, 103]. Cell death induced by p53 expression in yeast is Mca1p independent and mainly mediated by Nuc1p, suggesting the importance of yeast mitochondria in p53-induced cell death [104, 105]. Very recent studies have shown that the expression of p53 family proteins in yeast causes growth inhibition, increased actin expression and actin depolarization, ROS production and an autophagic cell death [21]. The expression in yeast of the well-known breast tumor suppressor genes BRCA1 and BRCA2 suggested a role of these genes in the maintenance of genomic stability and resulted in pleiotropic phenotype, such as the inhibition of cell growth and the formation of small colonies [101]. More recently, it has been reported that BRCA2 expression sensitizes yeast cells to acetic acid-induced programmed cell death [106].

## External triggers

Many external triggers have been shown to induce apoptosis in budding yeast including hydrogen peroxide, acetic acid, ethanol, high salt, osmotic stress, lipids, UV irradiation, heat stress, and numerous heavy metal ions [11].

### Acetic acid-induced cell death

A detailed description of the cellular events accompanying yeast cell death during exposure to acetic acid has been recently reported [107]. Acetic acid is one of the main sub-products, which accumulate in the culture medium during yeast alcoholic fermentation. Although *S. cerevisiae* can utilize acetate as carbon source, high concentration of acetic acid induces cell death (referred as AA-PCD) accompanied by all apoptotic hallmarks. In yeast, as in mammalian cells, mitochondria play a primary role in AA-PCD. In the course of exposure to acetic acid, yeast cells show mitochondrial swelling [108], membrane depolarization, ROS production, reduction of cytochrome oxidase activity and mitochondrial outer membrane permeabilization (MOMP), accompanied by release of cytochrome *c* and Aif1p [33, 109, 110]. The ROS scavenger N-acetylcysteine (NAC) prevents AA-PCD in wild-type cells while not in cells lacking Yca1p and cytochrome *c*. In these mutants, acetic acid can still induce cell death, although at lower rate compared to the respective wild-type strains, suggesting the existence of Yca1p and ROS-independent pathways [111, 112].

The partial prevention of AA-PCD by disrupting cytochrome *c* could be in relation with the increased mitochondrial membrane potential and with the lack of cytochrome *c* oxidase activity. According to this, respiratory deficient cells lacking mitochondrial DNA (ρ^0^ cells) display resistance to acetic acid-induced death [110]. AA-PCD is regulated by a number of proteins including Por1p (yeast voltage-dependent anion channel), which plays a protective role, and ADP/ATP carrier proteins, which are required for mitochondrial outer membrane permeabilization and cytochrome *c* release [113]. Other factors involved in some way in AA-PCD execution are Fis1p, Dnm1p and Mdv1p, the mitochondrial proteins responsible for fission and fusion events of these organelles, [114], and Pep4p, the yeast homologue of cathepsin D, which plays an important role for degradation of mitochondria in AA-PCD [43, 110]. Recently, it has been reported that components of the MAPK pathways modulate acetic acid-induced cell death. In fact, mutants in components of the mating pheromone response, as well in components of the high osmolarity glycerol (HOG) and the cell wall integrity (CWI) pathways are significantly more resistant to AA-PCD.

In addition, cytochrome *c* release from mitochondria was not detected in acetic acid-treated *bck1Δ* or *slt2Δ* mutants, indicating that the CWI pathway mediates acetic acid-induced apoptosis through a mitochondrial pathway [115].

It has been recently reported a proteomic study during AA-PCD in both wt and *mca1* null mutants indicating that in the absence of Yca1p cell death is induced through the activation of ceramides, whereas in the presence of the gene yeast cells underwent an AA-PCD pathway characterized by the shift of the main glycolytic pathway to the pentose phosphate pathway and by a proteolytic mechanism to cope with oxidative stress [116].

### Hyperosmotic stress

Hyperosmotic stress, an additional external trigger of cell death observed in mammalian cells, induces apoptosis in several pathological states such as diabetes, inflammatory bowel disease and hypernatremia [117]. Yeast can grow by fermentation in media containing up to 40 % glucose [118], a nutrient-rich but dangerous situation in that high osmolarity induces water efflux, cytosolic ions concentration and cell shrinkage. Following hyperosmotic stress, yeast activates adaptive responses that are mainly regulated by the high osmolarity glycerol (HOG) pathway, evolutionary conserved up to humans [119]. It has been reported that *S. cerevisiae* cells exposed to hyperosmotic stress in the presence of high glucose or sorbitol concentrations increase ROS production and show all peculiar apoptotic markers that require the involvement of mitochondria and of a partially *cyt c*-dependent Mca1p activation [120]. It has also been reported that high salinity, as high NaCl concentrations, induces in yeast apoptotic cell death [121] that follows Mca1p-dependent pathway in wild-type strains, while Mca1p-independent pathways in sro7/sro77 double mutants [122].

### Killer toxin

The “killer phenomenon” is widespread in different yeasts and consists of the secretion of protein toxins by killer strains that kill sensitive cells. Depending on the toxin nature, the killing mechanism is different in that some toxins act as ionophores disrupting the cytoplasmic membrane while others enter sensitive cells by receptor-mediated endocytosis, block DNA synthesis and arrest cell cycle in G1/S phase [123].

At low toxin concentrations, which are closer to the natural environmental situation, killer toxins, whatever the mode of cell killing, induce ROS production and Mca1p-dependent apoptotic cell death. In contrast, yeast cells exposed to high toxin undergo necrotic cell death [124].

### Pheromone-induced cell death

Yeast pheromones are short peptides secreted by cells of opposite mating types required for mating.

Pheromones, after binding to a G protein-coupled receptor (Ste2p or Ste3p), activate a specific MAP kinase signaling pathway allowing the induction of mating genes. The main component of the mating pathway is kinase Ste20p, the deletion of which prevents pheromone-induced death and the formation of ROS [125]. Pheromone-induced apoptosis takes place in consequence of increased intracellular Ca^2+^, ROS generation, formation of the permeability transition pore, Ysp1p-dependent mitochondrial fragmentation and release of cytochrome *c* [125, 126].

When cells are exposed to elevated concentrations of pheromone, as well after failure of mating, cell death seems to occur without showing certain peculiar apoptotic markers in a metacaspase-independent way, suggesting the activation of necrosis-like pathways [31]. This could represent an altruistic kind of death for the elimination of infertile or damaged haploid cells from the yeast community [11, 125].

### Effect of drugs on yeast cell death

The availability of gene deletion collection, together with the advancing development of yeast-based functional genomic and proteomic technologies, supports the utility of *S. cerevisiae* as a model organism in the drug-discovery process [127]. Due to the high conservation of basic cellular processes present in higher organisms, yeast is also a powerful system for the study of the mechanism of PCD-inducing drugs, since it is known that most of anticancer compounds induce apoptotic cell death.

Among antitumor drugs, the apoptotic phenotypes induced in yeast cells following paclitaxel, arsenic, bleomycin and valproate treatment, as well their mechanism of action, have been studied in detail. In the case of inhibitors of topoisomerases and/or cell cycle progression, there is not sufficient evidence for their role as RCD inducers, although many of them stimulate ROS production [128].

Some bioisosteres of arylthioindoles, which are potent tubulin assembly inhibitors, arrest growth of yeast and MCF-7 human breast carcinoma cells. Interestingly, the inhibition of tubulin polymerization in yeast triggers apoptosis mainly through *MCA1* and EndoG [129, 130].

Free fatty acids, in dependency on the degree of unsaturation, as well as ceramide, stimulate yeast cells to undergo regulated necrosis, [131]. Due to its sensitivity to most of antifungal drugs, yeast is used for the search and characterization of new compounds with lower toxicity against human pathogens. Molecules of different origins and mode of action, such as amphotericin B, osmotin, dermaseptin, pradimicin and histatin, exert their antifungal activity primarily through induction of apoptosis in *Saccharomyces cerevisiae* and/or *Candida albicans* [128].

The yeast model system can be applied also to study the beneficial effects of a range of molecules and nutrients on cells and large-scale search for anti-apoptotic molecules has been performed by the use of *fzo1* yeast mutants, which are impaired in mitochondrial fusion [132]. Some compounds such as acetyl-l-carnitine (ALC), used as therapeutic for stroke, myocardial infarction and neurodegenerative diseases, prolongs yeast lifespan in the presence of Mca1p, functional mitochondria and counteracts apoptosis [133]. Ascorbic acid, beta-carotene and caffeic acid, contained in some nutrients, are known antioxidants that reduce accumulation of ROS-protecting cells from aging and H_2_O_2_-induced cell death [134]. Apple’s extracts, rich in ascorbic acid and polyphenols are known to act as antitumors and prevent ROS accumulation, aging and cell death in yeast cells [135]. Resveratrol is a natural polyphenol, present especially in grapes, red wine and berries, showing antiaging and potential cardioprotective effects. This compound is also a potent inducer of autophagy through the activation of deacetylase SIRT1 in humans, as well of its ortholog *SIR2* in yeast. Spermidine, a polyamine found in citrus fruit and soybean, is an acetylase inhibitor and also an inducer of autophagy independent of human SIRT1 and yeast *SIR2* genes. It has been reported that both compounds induce autophagy by distinct pathways converging on the acetylproteome [136]. In yeast, deacetylation of histone H3 by spermidine reduces ROS levels produced during aging and has an anti-necrotic role promoting cell survival through the induction of autophagy [137].

## Organelles

### Mitochondria

Mitochondria play a central role in cellular functions including energy production, iron–sulfur biogenesis, calcium homeostasis, and RCD in eukaryotes. Most of the mitochondrial functions depend on the maintenance of tubular network deriving from the equilibrium between fusion and fission events. Mitochondrial fragmentation into punctuate structures is a common early feature of apoptosis in both mammalian and yeast cells [108, 138]. Apoptotic signals determine the release from the mitochondria of pro-apoptotic proteins such as cytochrome *c*, *AIF1*, EndoG and Omi/HtrA2 [138]. The release of cytochrome *c* from mitochondria, one of the signals triggering cell death, was firstly demonstrated in cells expressing the human pro-apoptotic protein Bax [139]. *AAC1/2/3* and *POR1* are the yeast orthologs of mammalian adenine nucleotide translocase (ANT) and voltage-dependent anion channel protein (VDAC), respectively. Their role in the release of cytochrome *c* during Bax-induced cell death is controversial, depending on the experimental conditions [140–142]. The absence of ADP/ATP carrier (AAC) proteins, encoded by AAC1/2/3, protected cells exposed to acetic acid and diamide but not to H_2_O_2_, while deletion of *POR1* enhances apoptosis triggered by all these compounds [113].

The overexpression of the human gene VDAC1 induces the accumulation of ROS and is toxic for yeast cells growing on respiratory carbon sources but not on glucose [143]. This could be explained by the increase within cell population of respiratory deficient mutants, which are known to show extended lifespan [66, 144]. The machinery responsible for mitochondrial fission in healthy yeast cells was identified as a complex of three proteins, Dnm1p, Mdv1p/Net2p, and Fis1p [145]. Dnm1p has been shown to promote mitochondrial fission and cell death following exposure to environmental stress while its deletion protects cells from death and aging [114, 146, 147].

Other proteins, involved in mitochondrial functions, protect cells from death. As an example, the absence of Cit1p, the major mitochondrial citrate synthase, or Isc1p, the inositolphosphosphingolipid phospholipase C, results in oxidative stress hypersensitivity associated with apoptotic markers that were suppressed by the contemporary absence of the Mca1p metacaspase [29, 148].

### Peroxisomes

Among cellular organelles, peroxisomes perform fundamental metabolic pathways such as β-oxidation of fatty acids and hydrogen peroxide detoxification.

Peroxisomes are ubiquitous in eukaryotes varying in shape, size and number adapting to cellular requirements. Like mitochondria, peroxisomes increase in their number by a fission mechanism, which in yeast requires Dnm1p or Vps1p, and it has been recently reported that the inhibition of peroxisome fission increases yeast chronological lifespan [149].

Together with mitochondria, peroxisomes are the main source of reactive oxygen species that accelerate aging and cell death, but their role in these processes still requires elucidation. Peroxisomes are involved in regulation of yeast necrosis. In fact, it has been reported that the deletion of *PEX6*, the gene encoding a protein involved in peroxisomal protein import, results in increased ROS production and loss of viability upon acetic acid treatment and during early stationary phase. Moreover, cell death in aging yeast cells lacking *PEX6* is not dependent on Mca1p and Aif1p and shows necrotic rather than apoptotic markers. The exact role of Pex6p in cells during acetic acid stress, as well in aging cells, remains to be clarified [150]. Interestingly, the deletion of the peroxisomal peroxiredoxin *PMP20* gene in the yeast *Hansenula polymorpha* results in enhanced ROS production and accumulation of lipid peroxidation in methanol growing cells. Similar to the scenario observed in mitochondria-mediated apoptosis, the absence of Pmp20p leads to loss of peroxisome membrane integrity with the release of matrix proteins into the cytosol followed by necrotic cell death [151].

### Endoplasmic reticulum (ER)

The yeast ER, as in all eukaryotes, is required for many relevant cellular functions such as the translocation and folding of proteins, the synthesis of lipids and the homeostasis of calcium.

In mammalian cells, loss of ER function leads to ER stress, which in turn can trigger endoplasmic reticulum stress-associated cell death (ER-SAD) [152]. Overload of proteins, exposure to long-chain saturated fatty acids, alterations in calcium levels and disturbances to the redox balance have been reported as the main factors leading to ER stress [153, 154]. The unfolded protein response (UPR) is a signaling network comprising three or two pathways in animal and plant cells, respectively. The only UPR pathway in yeast is represented by inositol-requiring protein-1 (*IRE1*), complementary to *IRE1* of plants where it plays an essential role in viral infection [155]. The UPR restores ER homeostasis by degrading misfolded proteins, by increasing levels of chaperones to improve protein folding and by inhibiting translation. Failure of UPR activation leads cells to death. The overexpression of Yno1p, an NADPH oxidase localized in the perinuclear ER belonging to the NOX superfamily able to produce superoxide (O_2_·−), induces yeast cell death dependent on the presence of Mca1p [156].

## Conclusions and perspectives

**Fig. 1:**
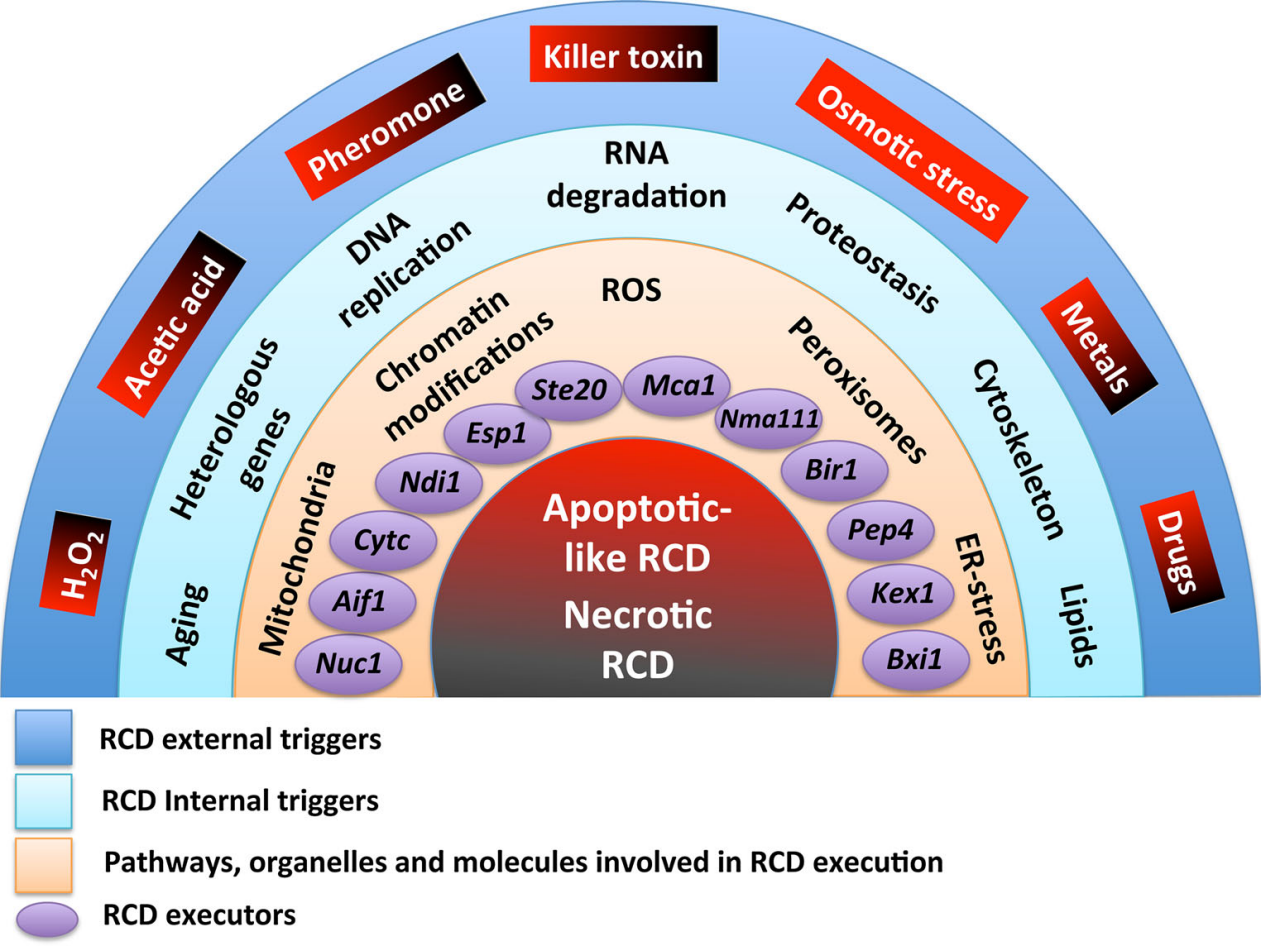
Regulated cell death (RCD) scenarios in yeast at glance. Apoptotic-like and necrotic RCD are represented in *red* and *black*, respectively. External triggers, included in *boxes* shading from *red* to *black*, induce apoptotic (*red*) or necrotic (*black*) RCD depending on their concentration

The current scenario of cell death has become more and more complex following the discovery of new forms of this process. The existence of the basal machinery of apoptosis, necrosis and apparently of autophagic cell death in yeast, makes this microorganism an easy model for the study of these phenomena. Figure 1 summarizes the actual scenario of regulated cell death.

Due to the easy manipulation by classical and molecular genetic approaches, yeast may be useful for the identification of new mammalian regulators and executors of cell death and, in the opposite direction, for the study of the effects of human genes in promoting or counteracting the different forms of RCD.

Finally, humanized yeast strains, expressing human genes not present in the yeast genome, constitute very powerful models for the study of aging, tumor progression, neurodegenerative disorders and for the development of new diagnostic assays and therapeutic molecules.
